# Comparative analysis of newest-generation variable-diameter cryoballoon with pulsed-field ablation for the treatment of atrial fibrillation: the CRYO-PULSE Study

**DOI:** 10.1093/europace/euaf166

**Published:** 2025-08-07

**Authors:** Behnam Subin, Corinne Isenegger, Felix Mahfoud, Michael Kühne, Sven Knecht, Philipp Krisai, Nicolas Schaerli, Merwan Wahab, Jan Per Wenzel, Charlotte Eitel, Christian-Hendrik Heeger, Karl-Heinz Kuck, Christian Sticherling, Roland Tilz, Patrick Badertscher

**Affiliations:** Department of Cardiology, University Hospital Basel, Basel 4031, Switzerland; Cardiovascular Research Institute Basel, University Hospital Basel, Petersgraben 4, Basel 4031, Switzerland; Department of Rhythmology, University Hospital Schleswig-Holstein, Lübeck 23562, Germany; Department of Cardiology, University Hospital Basel, Basel 4031, Switzerland; Cardiovascular Research Institute Basel, University Hospital Basel, Petersgraben 4, Basel 4031, Switzerland; Department of Cardiology, University Hospital Basel, Basel 4031, Switzerland; Cardiovascular Research Institute Basel, University Hospital Basel, Petersgraben 4, Basel 4031, Switzerland; Department of Cardiology, University Hospital Basel, Basel 4031, Switzerland; Cardiovascular Research Institute Basel, University Hospital Basel, Petersgraben 4, Basel 4031, Switzerland; Department of Cardiology, University Hospital Basel, Basel 4031, Switzerland; Cardiovascular Research Institute Basel, University Hospital Basel, Petersgraben 4, Basel 4031, Switzerland; Department of Cardiology, University Hospital Basel, Basel 4031, Switzerland; Cardiovascular Research Institute Basel, University Hospital Basel, Petersgraben 4, Basel 4031, Switzerland; Department of Cardiology, University Hospital Basel, Basel 4031, Switzerland; Cardiovascular Research Institute Basel, University Hospital Basel, Petersgraben 4, Basel 4031, Switzerland; Department of Rhythmology, University Hospital Schleswig-Holstein, Lübeck 23562, Germany; Department of Rhythmology, University Hospital Schleswig-Holstein, Lübeck 23562, Germany; German Center for Cardiovascular Research (DZHK), Partner Site Hamburg/Kiel/Lübeck, Lübeck, Germany; Department of Rhythmology, University Hospital Schleswig-Holstein, Lübeck 23562, Germany; German Center for Cardiovascular Research (DZHK), Partner Site Hamburg/Kiel/Lübeck, Lübeck, Germany; Department of Rhythmology, University Hospital Schleswig-Holstein, Lübeck 23562, Germany; German Center for Cardiovascular Research (DZHK), Partner Site Hamburg/Kiel/Lübeck, Lübeck, Germany; Department of Rhythmology, Asklepios Klinik Altona, Hamburg 22763, Germany; Department of Rhythmology, University Hospital Schleswig-Holstein, Lübeck 23562, Germany; Department of Cardiology, University Hospital Basel, Basel 4031, Switzerland; Cardiovascular Research Institute Basel, University Hospital Basel, Petersgraben 4, Basel 4031, Switzerland; Department of Rhythmology, University Hospital Schleswig-Holstein, Lübeck 23562, Germany; German Center for Cardiovascular Research (DZHK), Partner Site Hamburg/Kiel/Lübeck, Lübeck, Germany; Department of Cardiology, University Hospital Basel, Basel 4031, Switzerland; Cardiovascular Research Institute Basel, University Hospital Basel, Petersgraben 4, Basel 4031, Switzerland

**Keywords:** Atrial fibrillation, Pulsed-field ablation, Cryoballoon ablation, Pulmonary vein isolation, Catheter ablation

Pulmonary vein isolation (PVI) remains a cornerstone of atrial fibrillation (AF) treatment. Single-shot ablation devices, including cryoballoon ablation (CBA) and pulsed-field ablation (PFA), have simplified procedures while maintaining high efficacy.^1,2^ Recently, a variable-diameter cryoballoon (POLARx™ FIT) was introduced to improve anatomical conformity.^3,4^ Concurrently, PFA has emerged as a non-thermal, tissue-selective modality associated with favourable safety profiles.^5^

Direct comparisons between the newest-generation CBA and PFA technologies are limited. We present data from the CRYO-PULSE study, a prospective, multicentre analysis comparing procedural characteristics, safety, and mid-term efficacy of PFA vs. variable-diameter CBA in patients undergoing PVI.

This investigator-initiated study enrolled consecutive patients undergoing PVI for symptomatic AF at two tertiary centres (Basel, Switzerland, and Lübeck, Germany) between January 2022 and December 2024. After identifying the first 100 patients treated with POLARx™ FIT, 200 patients treated with the pentaspline PFA catheter (FARAPULSE™) were selected via 1:2 propensity score matching (age, sex, BMI, AF type, heart failure, hypertension, prior MI). Procedures were performed under conscious sedation. No 3D mapping was used for CBA. The primary endpoint was 12-month freedom from atrial arrhythmia (AF/AT > 30 s) after a 90-day blanking period. Follow-up included clinic visits with ECG/Holter at 3, 6, and 12 months.

A total of 300 patients (median age 67 years, 35% female, 52% paroxysmal AF) were included. After matching, baseline characteristics were largely balanced, except for coronary artery disease (28% CBA vs. 10% PFA, *P* < 0.001) and left ventricular ejection fraction (55% vs. 58%, *P* = 0.005). Antiarrhythmic drug use was slightly higher in the CBA group (32% vs. 18%, *P* = 0.004).

Median total procedure time was significantly shorter in the PFA group [47 (37–58) min] compared to CBA [52 (43–65) min; *P* = 0.003]. Fluoroscopy time was slightly longer in the PFA group [10 (8–13) min vs. 8 (6–12) min, *P* < 0.001]. First pass isolation was achieved more frequently with PFA (87% vs. 63%, *P* < 0.001). Median number of applications per patient was 34 (IQR 33–37, PFA) vs. 4 (4–5, CBA). Five complications occurred overall (1.7%). In the CBA group, one pericardial tamponade and one transient phrenic nerve palsy were recorded. In the PFA group, one tamponade, one transient ischaemic attack, and one stroke occurred. All patients recovered without lasting deficits.

At 12 months, arrhythmia-free survival was similar between groups: 77% (CBA) vs. 72% (PFA), *P* = 0.777 (*Figure 1*). Recurrence rates were comparable for paroxysmal and persistent AF subgroups. A total of 30 patients (10%) underwent redo ablation. Durable PVI was more frequently observed in the PFA group (39% vs. 14%; *Figure 1*).

**Figure 1:**
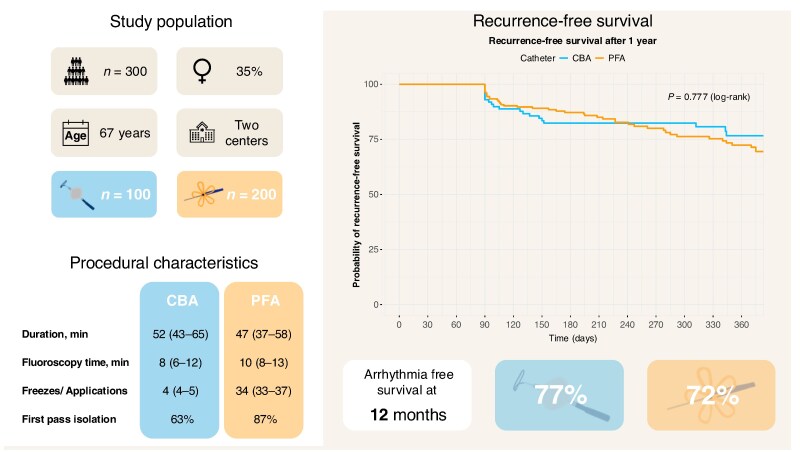
Study overview, procedural characteristics, and arrhythmia-free survival at 12 months.

This prospective, multicentre study is one of the first to compare the latest-generation variable-diameter CBA with a pentaspline PFA system for PVI. Pulsed-field ablation was associated with shorter procedure duration and higher first-pass isolation rates, while both technologies achieved comparable arrhythmia-free survival (∼70% at 12 months) and demonstrated favourable safety profiles. These findings are consistent with prior studies, including the ADVENT and MANIFEST-17 K studies,^1,2,5^ suggesting that PFA does not offer superior efficacy but improves procedural efficiency. Use of 3D-EAM in PFA cases may enhance lesion precision, though it is not essential for routine use. Fluoroscopy duration was longer in the PFA group, likely due to learning curve effects. Safety outcomes aligned with published registry data.^5,6^ Cost-effectiveness considerations favour shorter procedure times with PFA but are offset by higher device costs.^7^

Limitations include the observational design, lack of continuous rhythm monitoring, and absence of intracardiac echocardiography during PFA procedures. While this study focused on the pentaspline PFA catheter, other balloon-based PFA systems are emerging.^8^ Comparative studies are warranted. Future work should also address cost-effectiveness, long-term durability, and standardized lesion assessment across technologies.

In conclusion, in this prospective, matched analysis, PFA showed superior procedural efficiency with similar safety and mid-term clinical outcomes compared to variable-diameter CBA. These findings reinforce the need for randomized comparisons between current single-shot ablation technologies.

## Data Availability

The data underlying this article will be shared on reasonable request to the corresponding author.
